# Supplementary material for: Integrative genetic and genomic networks identify microRNA associated with COPD and ILD
Source: Sci Rep. 2023 Aug 11;13:13076. doi: 10.1038/s41598-023-39751-w (PMC10421936; doi:10.1038/s41598-023-39751-w)
Supplement: Supplementary file 1 — Supplementary Figures. [file 41598_2023_39751_MOESM1_ESM.docx]

**Integrative genetic and genomic networks identify microRNA associated with COPD and ILD**

Ana B. Pavel^1,2*^, Carly Garrison^1^, Lingqi Luo^1^, Gang Liu^1^, Daniel Taub^1^, Ji Xiao^1^, Brenda Juan-Guardela^3^, John Tedrow^3**^, Yuriy O. Alekseyev^4^, Ivana V. Yang^5^, Mark W. Geraci^5***^, Frank Sciurba^3^, David A. Schwartz^5^, Naftali Kaminski^3****^, Jennifer Beane^1,2^, Avrum Spira^1,2^, Marc E. Lenburg^1,2,4^, Joshua D. Campbell^1,2^

1. Department of Medicine, Boston University School of Medicine, Boston, MA, USA.

2. Bioinformatics Graduate Program, Boston University, Boston, MA, USA.

3. Department of Medicine, University of Pittsburgh Medical Center, Pittsburgh, PA, USA.

4. Department of Pathology and Laboratory Medicine, Boston University School of Medicine, Boston, MA, USA.

5. Department of Medicine, University of Colorado, Aurora, CO, USA.

* Now at Department of Biomedical Engineering, University of Mississippi, University, MS, USA.

** Now at St. Elizabeth's Medical Center-Brighton, Brighton, MA, USA.

*** Now at Department of Medicine, University of Pittsburgh Medical Center, PA, USA.

**** Now at Department of Medicine, Yale School of Medicine, New Haven, CT, USA.

Corresponding authors:

Joshua D. Campbell

Email: camp@bu.edu

72 East Concord St

Boston, MA 02118 USA

Ana B. Pavel

Email: apavel@olemiss.edu

303 Brevard Hall

University, MS 38655 USA

**SUPPLEMENTARY FIGURES**

**
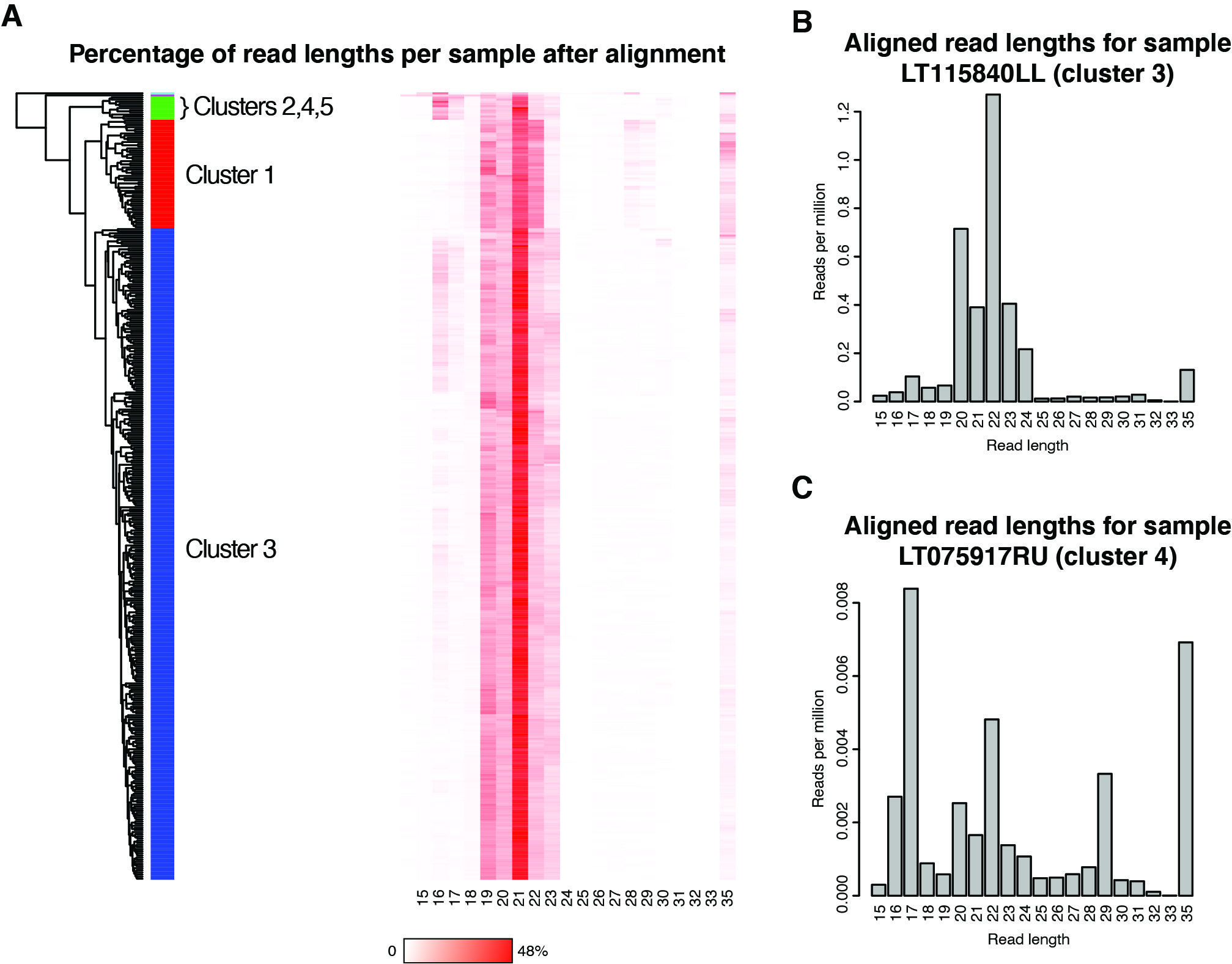
**

**Supplementary Figure 1. Sample filtering using the distribution of aligned read lengths.** The small RNA of 371 samples was sequenced and reads with a length less than 15 bases were excluded after trimming. **(A)** After alignment with Bowtie, samples were clustered on the normalized distribution of aligned read lengths using the Jensen-Shannon divergence as the distance metric and hierarchical clustering with average linkage. The 5 most distinct clusters were identified with the *cutree* function. The majority of samples fell into clusters 1 and 3. The distribution of aligned read lengths for samples in clusters 1 and 3 had a peak at 21 bases, which is expected as the small RNA fraction should largely contain miRNAs. Clusters 2, 4, and 5 had aligned read length distributions with peaks at other bases and were excluded from the down-stream analysis. **(B)** LT115840LL is a representative sample of cluster 3 which had expected read length distributions. **(C)** LT075917RU was in cluster 4 and had unexpected peaks at 17 and 29 bases and thus was excluded from the down-stream analyses.

**
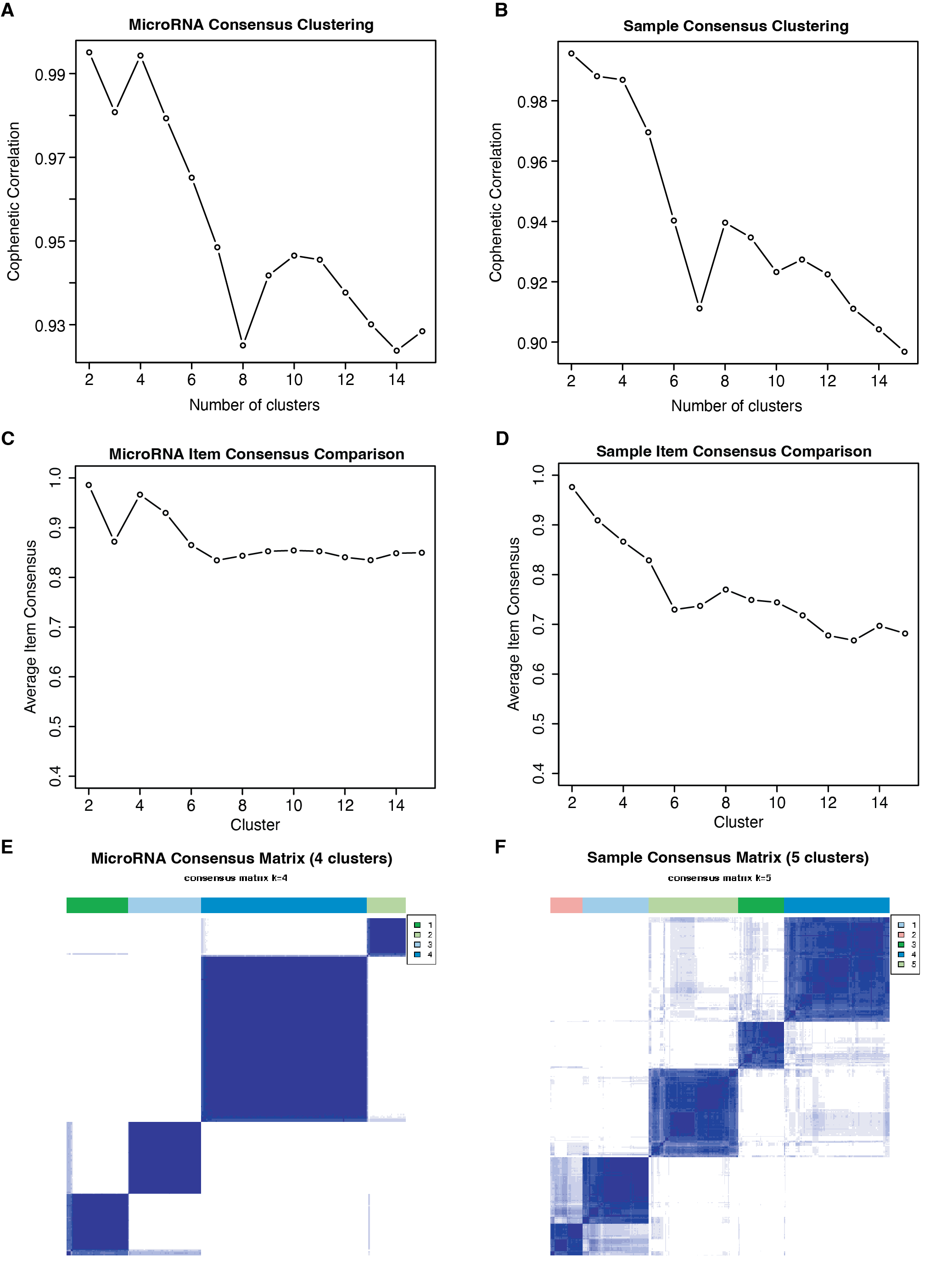
**

**Supplementary Figure 2. Consensus clustering of miRNAs and samples.** The *ConsensusClusterPlus* R package was used to separately cluster miRNAs and samples into group. The Cophenetic coefficient, Average Item Consensus, and Consensus Matrices were used to help determine the numbers of miRNA and sample clusters. **(A)** The k=4 solution was the most optimal number of clusters for the miRNA after k=2 for both the Cophenetic score and the Average Item Consensus. While a large drop could be observed in the scores from k=4 to k=5, only a relatively small number of miRNA (n=17) were split from the largest cluster. Furthermore, the two new miRNA clusters displayed similar patterns as the larger cluster from the k=5 solution. Therefore, we choose the k=4 solution for the miRNA clustering. MiRNA clusters 1, 2, 3, and 4 were renamed to M4, M2, M3, and M1, respectively, when displayed in the heatmap in **Figure 1**. **(B)** While the most optimal number of sample clusters was 2 for both the Cophenetic coefficient and Average Item Consensus, a large drop could be observed after the k=5 solution in both metrics. The k=5 solution also resulted in two distinct ILD-enriched clusters with different patterns of expression for miRNA module M2. Therefore, we choose the k=5 solution for the sample clustering. Samples clusters 1, 2, 3, 4, and 5 were renamed to S5, S4, S3, S1, and S2, respectively, when displayed in the heatmap in **Figure 1**.

**
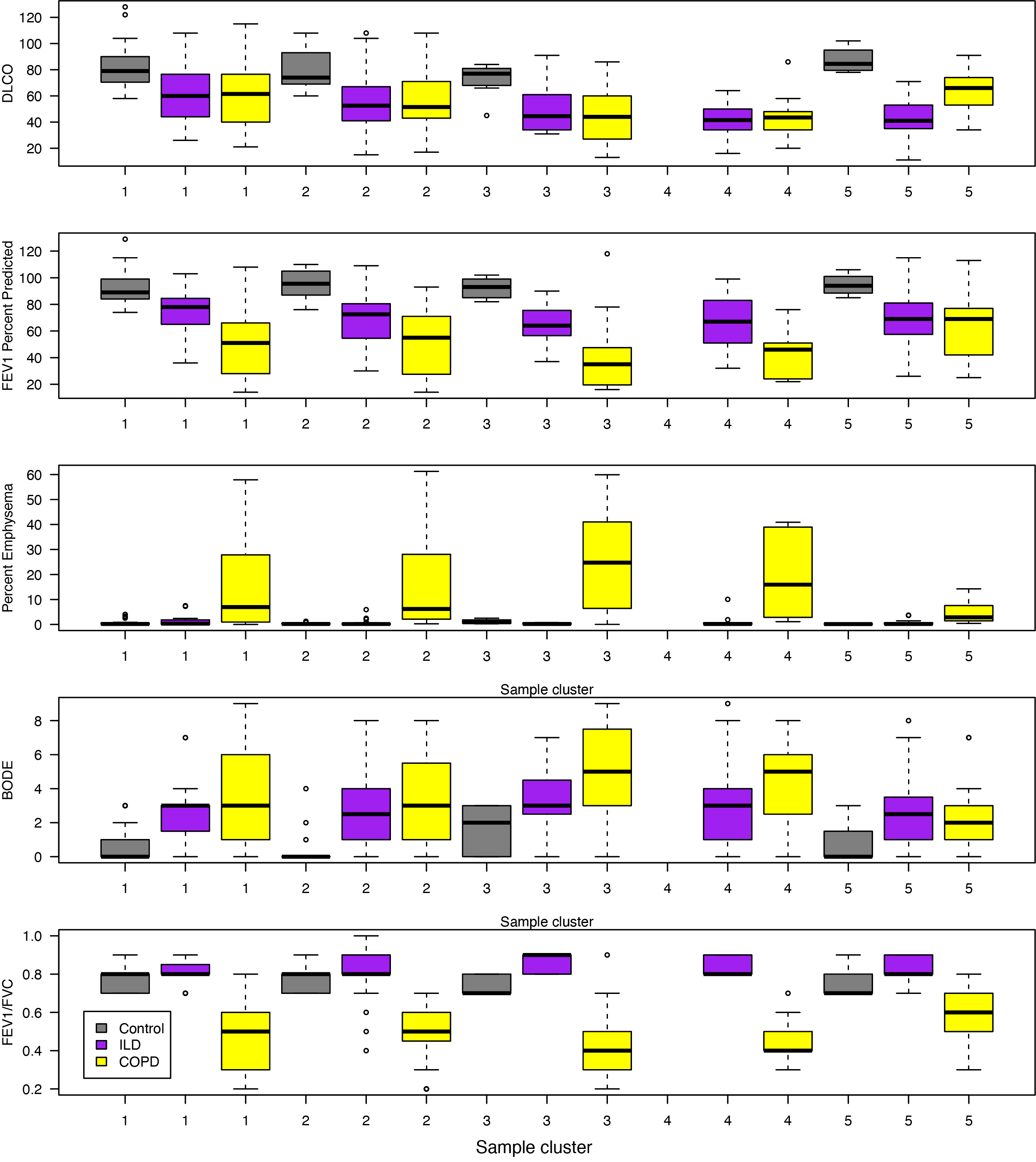
**

**Supplementary Figure 3. Distribution of clinical features across sample clusters and disease status.** In general, DLCO was similar between ILD and COPD within each sample cluster with the exception of sample cluster S5 in which ILD samples had lower DLCO compared to COPD samples. FEV1 Percent Predicted was lower in COPD patients compared to ILD and Control samples within each cluster with the exception of sample cluster S5 where ILD and COPD samples were similar. Similarly BODE was higher in COPD samples compared to ILD and Control samples with the exception of sample clusters S2 and S5 where ILD and COPD samples were similar. ILD patients had lower FEV1 Percent Predicted and higher BODE compared to Controls within each cluster with the exception of sample cluster S4 which did not have Control samples. FEV/FVC was lower for COPD samples compared to ILD and Control samples in each cluster but not different between ILD and Control samples. Percent Emphysema was higher in COPD samples in all clusters.

**
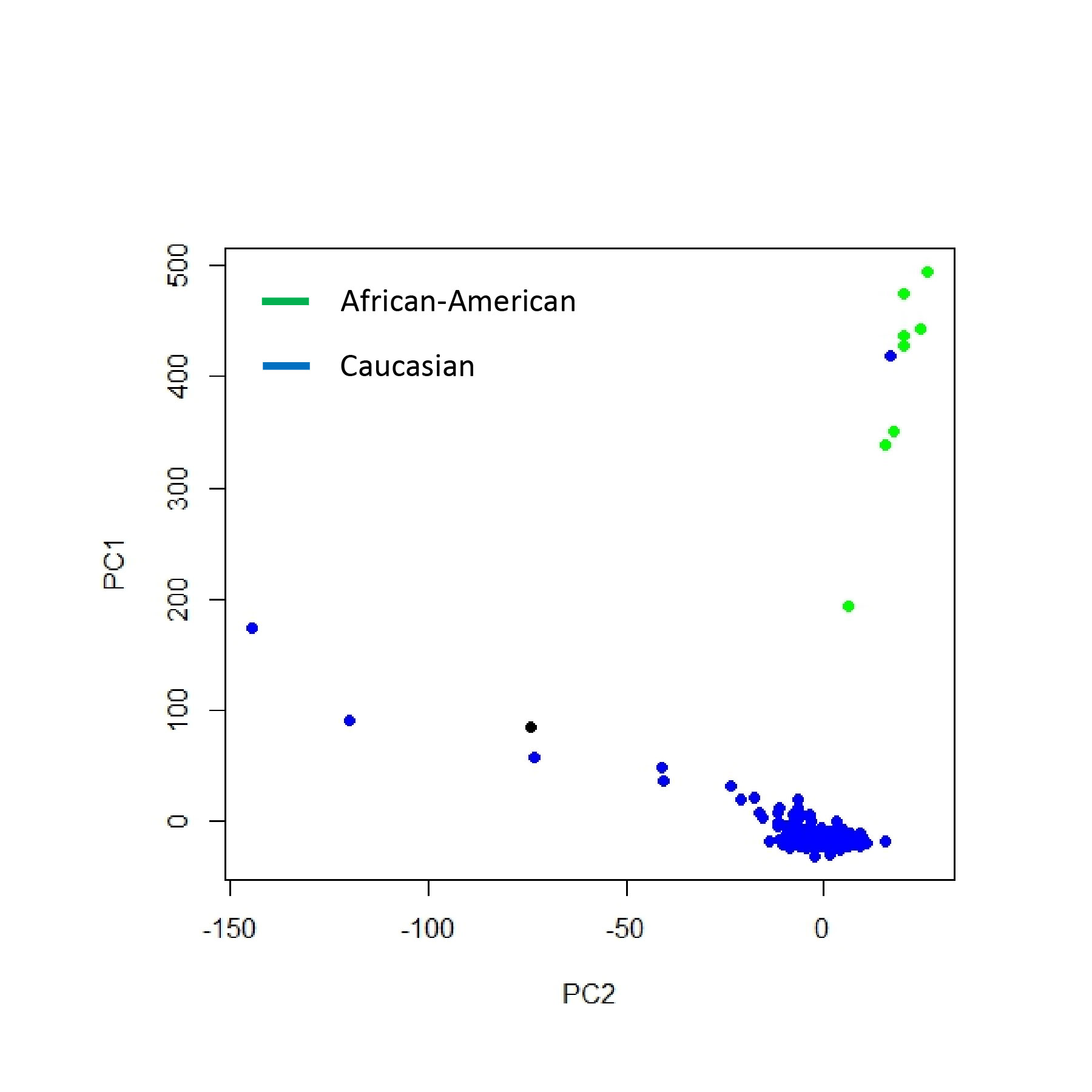
**

**Supplementary Figure 4. PCA plot showing the genetic population structure measured with the SNP chips.** Points are labeled by self-reported ethnicity.


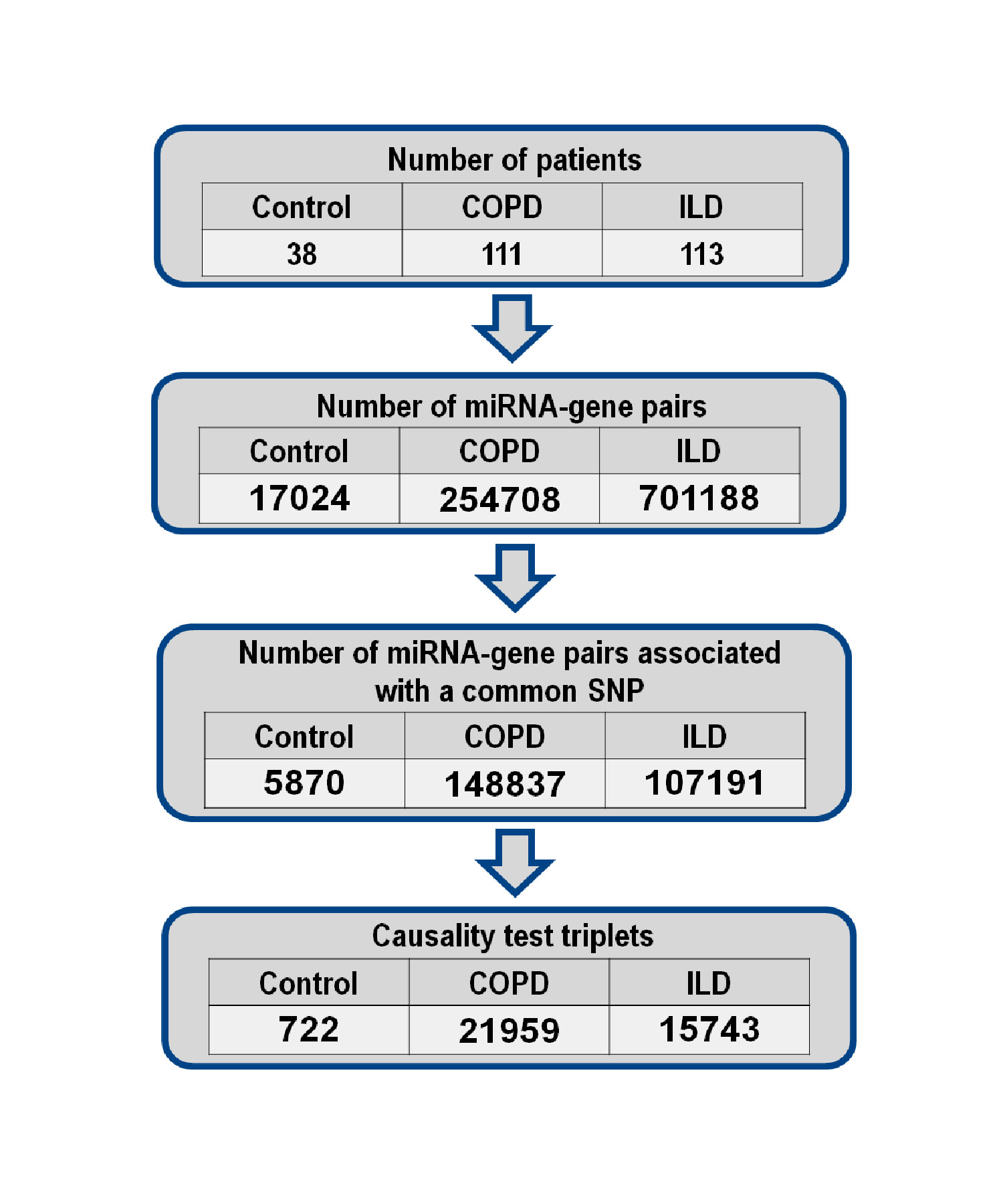


**Supplementary Figure 5.** **CIT network construction.** Number of significant interactions at each step of network construction in COPD, ILD and control groups.


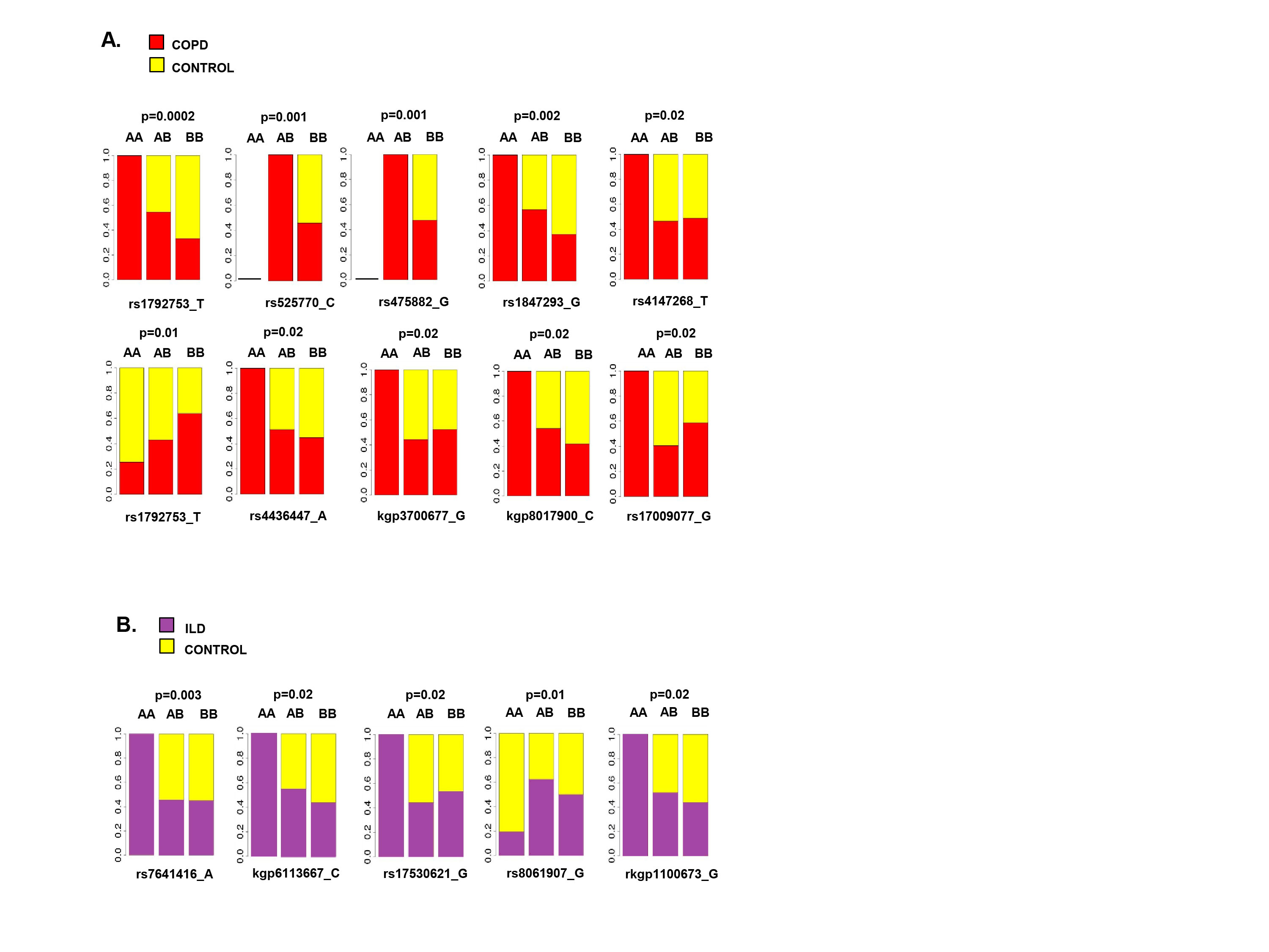


**Supplementary Figure 6.** **A.** SNPs that are significantly associated with COPD by a Fisher’s exact test (q<0.25). **B.** SNPs that are significantly associated with ILD by a Fisher’s exact test (q<0.25).

**
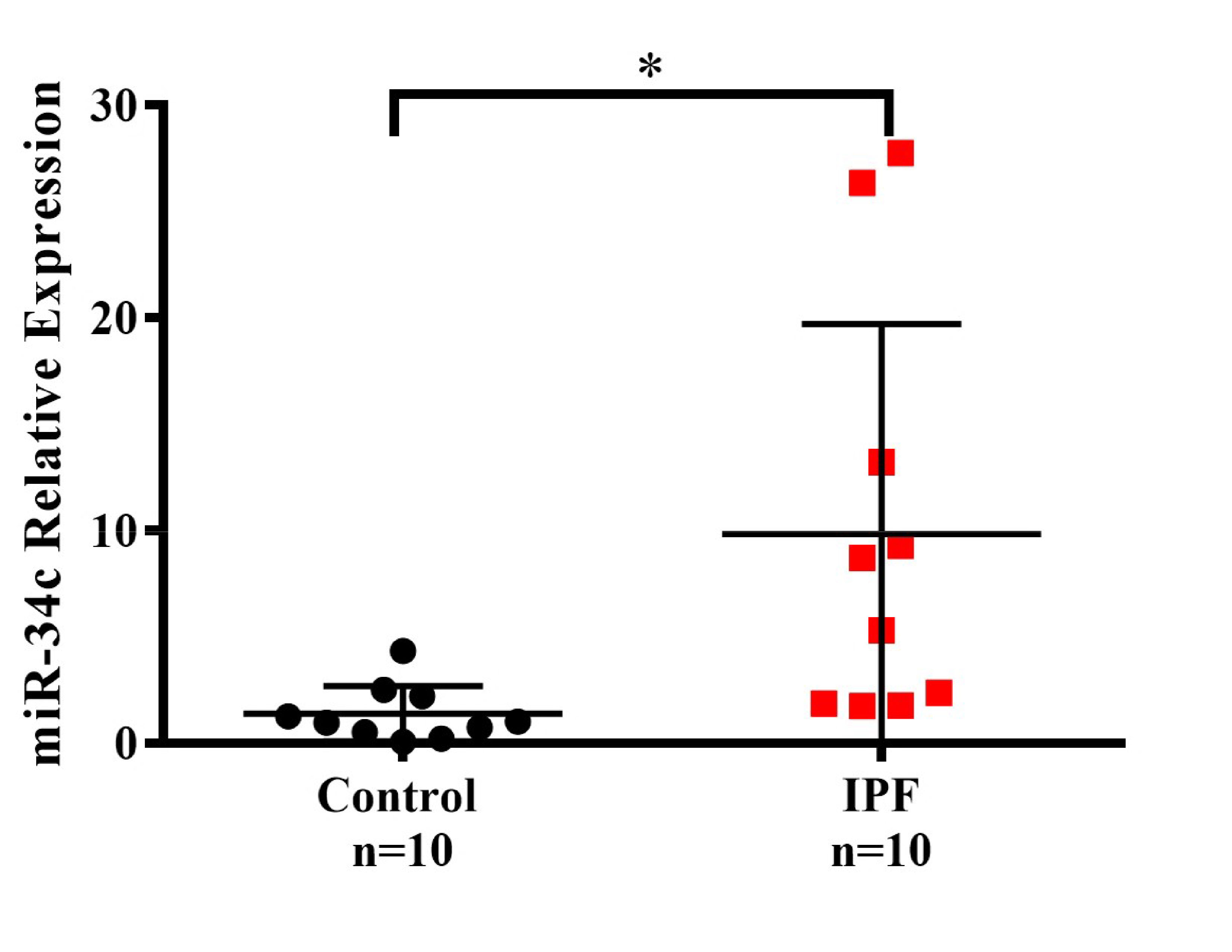
**

**Supplementary Figure 7.** Upregulation of miR-34c in ILD tissue validates by qRT-PCR. IPF (n=10) tissues have a significantly higher expression of miR-34c then control (n=10) tissues (p<0.05).

#
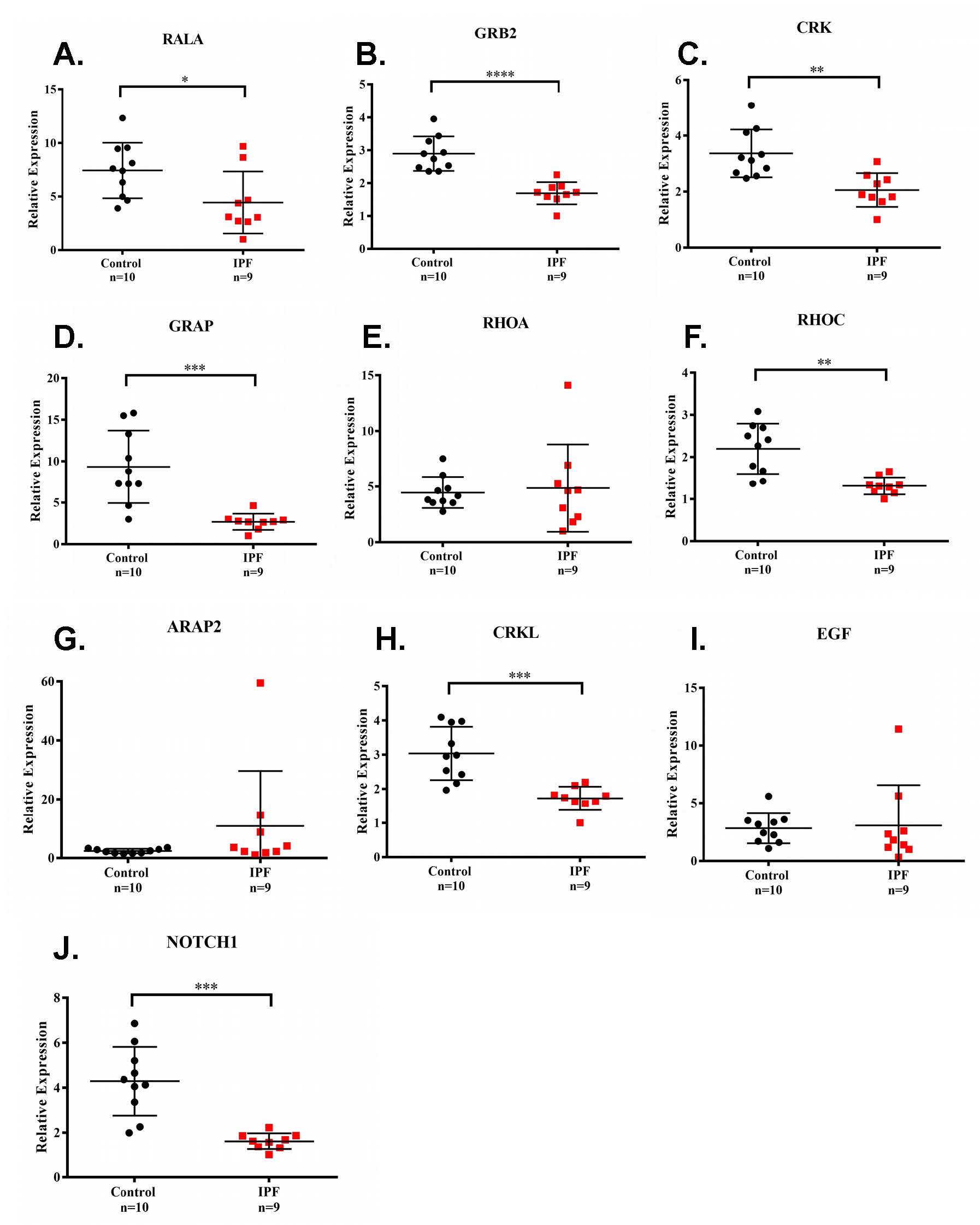


Supplementary Figure 8. Of the ten genes measured in clinical samples, nine of which are members of the Ras protein signaling pathway, seven validated as being downregulated in ILD compared to control tissue. These seven genes are: A. RALA (p<0.05), B. GRB2 (p<0.00005), C. CRK (p<0.005), D. GRAP (p<0.0005), F. RHOC (p<0.005), H. CRKL (p<0.0005), and J. NOTCH1 (p<0.0005).
